# Novel application of heuristic optimisation enables the creation and thorough evaluation of robust support vector machine ensembles for machine learning applications

**DOI:** 10.1007/s11306-015-0894-4

**Published:** 2015-11-21

**Authors:** Eleni Anthippi Chatzimichali, Conrad Bessant

**Affiliations:** School of Biological and Chemical Sciences, Queen Mary University of London, London, E1 4NS UK

**Keywords:** Multivariate classification, SVMs, Optimisation, Ensembles, Bootstrapping, Validation

## Abstract

Today’s researchers have access to an unprecedented range of powerful machine learning tools with which to build models for classifying samples according to their metabolomic profile (e.g. separating diseased samples from healthy controls). However, such powerful tools need to be used with caution and the diagnostic performance of models produced by them should be rigorously evaluated if their output is to be believed. This involves considerable processing time, and has hitherto required expert knowledge in machine learning. By adopting a constrained nonlinear simplex optimisation for the tuning of support vector machines (SVMs) we have reduced SVM training times more than tenfold compared to a traditional grid search, allowing us to implement a high performance R package that makes it possible for a typical bench scientist to produce powerful SVM ensemble classifiers within a reasonable timescale, with automated bootstrapped training and rigorous permutation testing. This puts a state-of-the-art open source multivariate classification pipeline into the hands of every metabolomics researcher, allowing them to build robust classification models with realistic performance metrics.

## Introduction

In many areas of biology, machine learning algorithms are used to build models to identify the type, or state, of biological samples from multivariate analytical data. Examples include diagnosis of cancer from vibrational spectra (Sattlecker et al. 2014), confirmation of food authenticity of milk and milk products (Nicolaou et al. 2011), and determination of food freshness (Argyri et al. 2013). The models produced by machine learning algorithms are essentially performing pattern recognition, sometimes referred to more formally as *multivariate classification*. In the metabolomics community such models have long been used to demonstrate that there is an objectively discernible biochemical difference between sample classes. This is often used to prove a hypothesis, but can also be considered as a first step towards automating the classification of unknown samples, or identifying biomarkers that could be used as the basis of a novel diagnostic test.

There is a large and growing list of machine learning methods available, including linear discriminant analysis (LDA) (Klecka 1980), partial least squares discriminant analysis (PLS-DA) (Wold et al. 2001; Barker and Rayens 2003), artificial neural networks (ANNs) (Hornik et al. 1989; McCulloch and Pitts 1943; Sanger 1989; Yegnanarayana 2009), random forests (Breiman 2001) and support vector machines (SVMs) (Boser et al. 1992; Cortes and Vapnik 1995). Within the metabolomics community, PLS-DA predominates to such an extent that some researchers are not fully aware of the alternatives (Thissen et al. 2004; Szymańska et al. 2012; Gromski et al. 2015). However, other approaches are now gaining ground, with SVMs in particular being successfully applied in metabolomics and beyond (Mahadevan et al. 2008; Liland 2011; Luts et al. 2010). One of the key features of SVMs, as opposed to traditional chemometrics techniques, is the support for both linear and nonlinear prediction models with boundaries of high complexity, which can satisfy the extremely complex nature of metabolomic data (Luts et al. 2010; Xu et al. 2006). Several direct comparisons between SVMs and PLS-DA have shown that SVMs can outperform PLS-DA in terms of prediction accuracy when applied to metabolomics data (Mahadevan et al. 2008; Thissen et al. 2004; Gromski et al. 2015).

Today, building a classification model using any of the aforementioned machine learning methods is technically straightforward thanks to readily available software implementations and an abundance of computing power (Ratner 2011). However, ascertaining a truly representative indication of the classification accuracy for the intended application can be a challenge, potentially leading non-experts to invalid conclusions (Domingos 2012). Overly optimistic assessments of performance are commonplace, leading to classification models that appear to work well in a pilot study often failing when applied to data from a new set of samples.

The most crucial step in supervised learning is the evaluation (testing) process where the generalisation performance of a classifier is assessed on previously unseen data (Geman et al. 1992; Wold et al. 2001; Izenman 2008). The first indicator frequently used to estimate the overall predictive power of a pattern recognition system is the classification accuracy (%CC), which is equal to the percentage of correctly classified samples. Metrics such as sensitivity and specificity, or in cases of multi-class studies the per class accuracies, provide further detail about classification model performance. However, like all performance metrics, the overall classification accuracy, sensitivity and specificity vary substantially according to how exactly the testing is performed. Most metabolomics practitioners are aware that testing a model on exactly the same data that was used to train it is inappropriate because it would lead to perfect training scores (i.e. sensitivities and specificities of 100 %) but would fail to predict new unseen data (Kohavi 1995). Testing with a second data set, totally independent of the training data, is the obvious solution to this problem but proves difficult when limited numbers of samples are available (as is often the case, particularly in clinical studies) and there is a danger of obtaining a fluke result because a single independent test set happens to give particularly good or bad results. This has led to the widespread use of cross-validation (Stone 1974) techniques where testing is performed using mutually exclusive subsets (folds) of the data with approximately equal size, the results of which are combined by averaging. However, cross-validation has been shown to substantially overestimate model performance due to instances of high variance (Kohavi 1995; Westerhuis et al. 2008). Bootstrapping (Efron 1979; Efron and Tibshirani 1994) is therefore the currently preferred solution, whereby new datasets (bootstrap samples) are created from the original data by randomly sampling with replacement. By repeating this resampling process a great number of times, a good estimate of the underlying sampling distribution (Wehrens et al. 2000) can be obtained. More specifically, one of the main advantages of bootstrapping is the fact that it allows robust evaluation of statistical properties (e.g. standard errors, confidence intervals, bias) that would be difficult to obtain analytically (Tichelaar and Ruff 1989; Massart et al. 1997; Wehrens et al. 2000; Liland 2011). However, we must still question whether the model performance obtained is significant compared to random chance. This final step is achieved using permutation testing (Good 2004), whereby the whole model building and testing process is repeated hundreds of times in an attempt to map samples to randomly permuted classes—a model performance that does not differ substantially from performance achieved for the random permutations cannot be considered significant.

From this brief explanation, it is clear that testing procedures have an overwhelming influence on the veracity of performance metrics calculated when applying machine learning and that performing the testing process properly can be laborious and computationally intensive. Indeed, training and rigorous evaluation for a single classification problem requires expert knowledge and can involve training millions of individual classifiers, which can be extremely computationally demanding especially if these classifiers involve complex models such as nonlinear SVMs. To address this issue, we have developed the *classyfire* R package for the implementation of ensemble SVM training with bootstrapping and rigorous performance evaluation via a handful of high-level functions. The key to this package is a novel solution for optimising SVM hyperparameters that bestows a speed up of more than tenfold compared to the widely applied grid search. We believe that making such a high quality multivariate classification pipeline readily available will improve the quality of metabolomics research by providing a transparent and trusted model building and evaluation workflow that can be used by researchers with limited machine learning experience and inexpensive computer hardware.

## Methods

### Support vector machines

SVMs were chosen for this work because of their proven ability to produce classification models that outperform equivalent PLS-DA models for many metabolomics applications. A detailed explanation of the theory behind SVMs is beyond the scope of this paper (such explanations can be found in Cortes and Vapnik (1995) and Cristianini and Shawe-Taylor (2000)) but, in summary, a SVM attempts to separate classes within the variable space by fitting a hyperplane between different sample groups in a way that produces a low generalisation error while simultaneously aiming to maximise the distance (margin) between the nearest points of the two classes (Bennett and Campbell 2000; Suykens et al. 2002). Because the complexity of most metabolomics datasets makes linear separation between classes impossible, a nonlinear kernel function is typically used to project the data into a higher dimensional feature space where linear separation is theoretically feasible (Chapelle and Vapnik 1999; Cristianini and Shawe-Taylor 2000). Common nonlinear kernels include the radial basis function (RBF, also called Gaussian), polynomial function and sigmoid function (Hearst et al. 1998). Each of these kernels is characterised by a set of hyperparameters that have to be carefully tuned for the specific problem under study (Chapelle et al. 2002). The radial basis function (RBF) kernel is particularly popular and a reasonable first choice (Hsu et al. 2003), especially in cases where there is little or no knowledge about the data under study. The optimisation of RBF SVMs requires the thorough tuning of two hyperparameters—the cost parameter *C*, which controls the optimal trade-off between maximising the SVM margin and minimising the training error, and the kernel parameter *γ* (gamma), which determines the degree of nonlinearity or width of the RBF kernel. Various methods have been devised to extend the binary classification functionality of SVMs to multi-class cases, usually by dividing a multi-class problem in a series of binary problems (Hsu and Lin 2002; Duan and Keerthi 2005).

### Bootstrap training of RBF SVMs

**Fig. 1:**
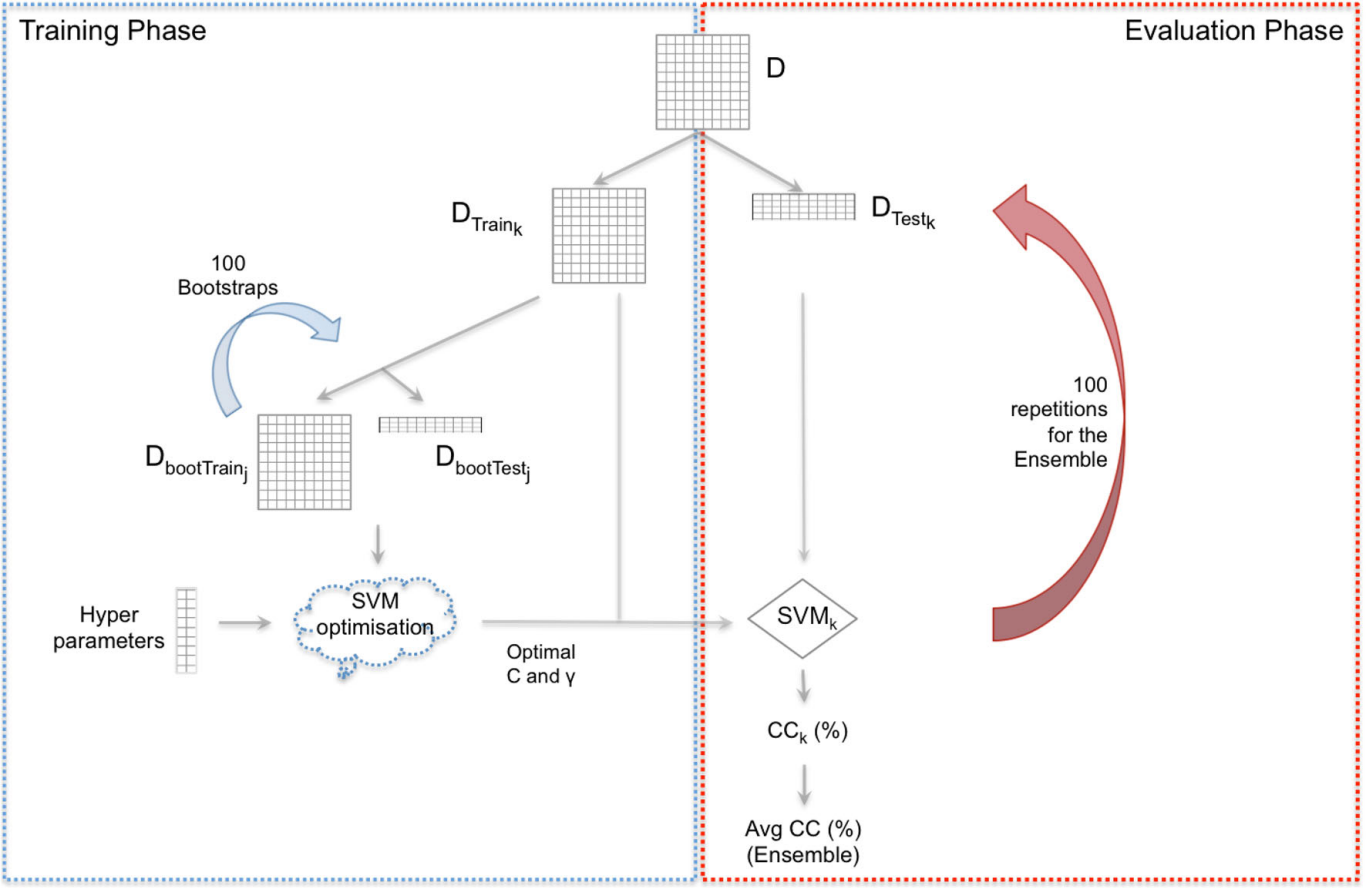
Flow diagram illustrating the overall process of constructing an ensemble of RBF SVMs optimised via boostrapping. The process is distinctly split into two steps—the training and testing (evaluation) process

As mentioned in the introduction, bootstrapping is currently the preferred method for validating classification models because it often gives more representative and robust performance metrics than other validation techniques (Wehrens et al. 2000; Liland 2011). Figure 1 demonstrates how we have implemented bootstrapping in our model building workflow. For a given input dataset *D*, a random fraction of samples is removed and kept aside as an independent test set during the training process of the model (holdout process). This selection of samples forms the dataset *D*_*test*_. This test set typically comprises a third of the original samples, therefore the test set consists of the same balance of sample classes as the initial dataset *D* (stratified holdout). The remaining samples that are not selected form the training set *D*_*train*_. Since the test set is kept aside during the whole training process, the risk of overfitting is minimised (Ramadan et al. 2006). In the case of bootstrapping, a bootstrap training set *D*_*bootTrain*_ is created by randomly picking $n$ samples with replacement from the training dataset *D*_*train*_. The total size of *D*_*bootTrain*_ is equal to the size of *D*_*train*_. Since bootstrapping is based on sampling with replacement, any given sample could be present multiple times within the same bootstrap training set. The remaining samples not found in the bootstrap training set comprise the bootstrap test set *D*_*bootTest*_. In the case of RBF models with bootstrapping, the SVMs are built and optimised using *D*_*bootTrain*_ and *D*_*bootTest*_ for different hyperparameter settings. More specifically, for each given combination of the hyperparameters *C* and *γ*, a new SVM model is trained with *D*_*bootTrain*_ and tested with *D*_*bootTest*_. To avoid reliance on one specific bootstrapping split, bootstrapping is repeated at least 100 times until a clear winning parameter combination emerges. Several methods can be used to determine the winning parameter; most commonly, the statistical average or the parameter that has most frequently been recorded as optimal is used.

### SVM optimisation and ensembles

The optimisation of the hyperparameters is traditionally implemented using a two-step approach based on a combination of a coarse and fine grid-search, where the SVM performance is evaluated at regular intervals across the *C*-*γ* surface (the ranges are set to *C* = [2^−5^, 2^−3^, …, 2^15^] and *γ* = [2^−15^, 2^−13^, …, 2^5^] respectively) and the best parameter combination used to seed a finer grid search to refine the values of *C* and *γ* (Hsu et al. 2003; Meyer et al. 2003). This is a relatively slow process, which becomes a particular hindrance when bootstrapping is used since many individual SVMs must be optimised. We have therefore implemented a much faster optimisation strategy based on a constrained nonlinear simplex optimisation (Box 1965), which performs the minimisation of the average bootstrapping test error during the training process of the SVMs within acceptable timescales. In this case, the inequality constraints correspond to the minimum and maximum predefined hyperparameter boundaries, where $\log_{2} \gamma \in \left\{ { - 15, 5 } \right\}$ and $\log_{2} C \in \left\{ { - 5, 15 } \right\}$. The formation of the initial complex begins with the selection of a random feasible point that must satisfy the minimum and maximum hyperparameter constraints. The simplex easily adapts itself to the local landscape such as a three-dimensional surface plot by elongating itself down long slopes, altering direction when encountering a valley at an angle, and contracting as it approximates the minimum (Singer and Nelder 2009). A thorough review and step-by-step explanation of the simplex methodology can be found in Nelder and Mead (1965), and Lagarias et al. (1998).

Ultimately, the optimal parameters are used to train a new classifier with the full *D*_*train*_ dataset and test it on the independent test set *D*_*test*_, which has been left aside during the entire optimisation process. Even though the approach described thus far generates an excellent classifier, the random selection of test samples in the initial split may have been fortunate. For a more accurate and reliable overview, the whole process is repeated a minimum of 100 times, as illustrated in Fig. 1, until a stable average classification rate emerges. The output of this repetition consists of at least 100 individual classification models built using the optimum parameter settings. At this stage, rather than isolating a single classification model, all individual classifiers are fused into a classification ensemble. Ensembles have repeatedly been shown to perform better than individual classifiers (Opitz and Maclin 1999; Dietterich 2000; Westerhuis et al. 2008) and have the added benefit of providing a measure of confidence in the predictions – the greater the number of models that vote for a reported class the more confident we can be that this class is correct.

### Calculation of performance metrics

The first indicator frequently used in multivariate classification is the percentage of correctly classified samples (%CC):

$$\% CC = \frac{{N_{c} }}{{N_{c} + N_{nc} }} \times 100\,\%$$

where $N_{c}$ and $N_{nc}$ are the number of correct and incorrect classifications respectively (Ciosek et al. 2005). The sum of $N_{c}$ and $N_{nc}$ is equal to the total number of instances $n$ in the dataset. The model with the maximum number of correctly classified samples is considered optimal.

In a similar manner, the percentages of correctly classified samples per class are also calculated. The comparison of the individual class predictions is important as the overall accuracies of a classifier may occasionally be misleading.

### Permutation testing

Nonparametric permutation testing can be applied as a means of providing an indication of the statistical significance of the classification model performance (Anderson 2001). In each permutation iteration, the input data matrix remains unaltered while the associated class vector is randomly shuffled; thus, the class distribution in the dataset remains unaltered, however, the samples correspond to randomly assigned classes. This procedure randomises the association between the input data and the classes, while their initial distributional properties are preserved (Westerhuis et al. 2008). Permutation testing is performed repeatedly a large number of times (usually a minimum of 100 times) until a stable distribution under the null hypothesis is obtained. In this case study, the null hypothesis that we are trying to reject assumes that there is no significant relationship between the observed data and the sample classes, and therefore a classification model could have been built to group samples into any arbitrary class.

At the end of permutation testing, we can determine the frequency of models that presented accuracies equal to or higher than the original model. A frequency metric commonly used when testing a statistical hypothesis is the $p$-value (Hubert and Schultz 1976). A $p$-value less than or equal to a predefined threshold value—commonly referred to as the significance level—indicates that the observed data are inconsistent with the assumption that the null hypothesis is true, and thus the null hypothesis must be rejected. A particular benefit of $p$-values is that they are directly comparable across different cases regardless the number of samples, variables and classes in a dataset. However, it is important to exercise caution when using *p*-values as a basis for biological conclusions as they are not as reliable or as objective as most scientists believe (Nuzzo 2014).

### R implementation

All of the above methods have been implemented in a new R package called *classyfire* (http://cran.r-project.org/package=classyfire). This implementation is highly integrated, such that most of the functionality is accessed using just three functions. The *cfBuild()* function implements the training and testing workflow as outlined in Sects. 2.2-2.4. As a minimum, two objects need to be provided as input to the workflow. One of these is the data matrix containing the data associated with every sample under study. Any alignment or other pre-processing must be applied prior to passing the data to the function. The other mandatory input object contains essential information about the experimental design, specifically the group (class) to which each sample belongs. Optional objects are used to configure specific details of the workflow, such as the number of ensembles and bootstrap iterations to perform as well as arguments that determine whether execution is in sequence or in parallel.

On completion of the workflow, the function outputs an object containing the classification ensemble produced, together with detailed performance metrics. This object can be used to classify samples in further datasets using the *cfPredict()* function, and can also be interrogated to reveal performance metrics, in both numeric and graphical forms. The *cfPermute()* function is used to perform permutation testing to indicate the statistical significance of the classification performance obtained, as described in Sect. 2.5.

### Datasets used

To demonstrate the use of the *classyfire* package, it was applied to two well-understood NMR datasets, both of which are included in the publicly available *MetabolAnalyze* R package (http://cran.r-project.org/package=MetabolAnalyze). These are simulated datasets designed to mimic experimental data previously reported in Carmody and Brennan (2010), and Nyamundanda et al. (2010). In brief, mice were randomly assigned to two treatment groups and treated with pentylenetetrazole (treated group) or saline (control group) for a period of 4 weeks. Urine was collected and at the end of the treatment period brain regions were isolated and metabolites extracted, and all samples analysed using NMR. In the following, dataset A is used to refer to the urine dataset—an 18 sample, 189 variable (189 spectral bin) dataset, which is split 50/50 between the two treatment groups (treated *vs* control). Dataset B is the brain dataset, comprising 33 samples (all from the control group mice) of 164 variables with spectra collected from four different areas of rat brain: brain stem, cerebellum, hippocampus and pre-frontal cortex (Nyamundanda et al. 2010). These datasets therefore provide an example of a two class problem and a four class problem respectively.

## Results

### Evaluation of classification accuracy

**Fig. 2:**
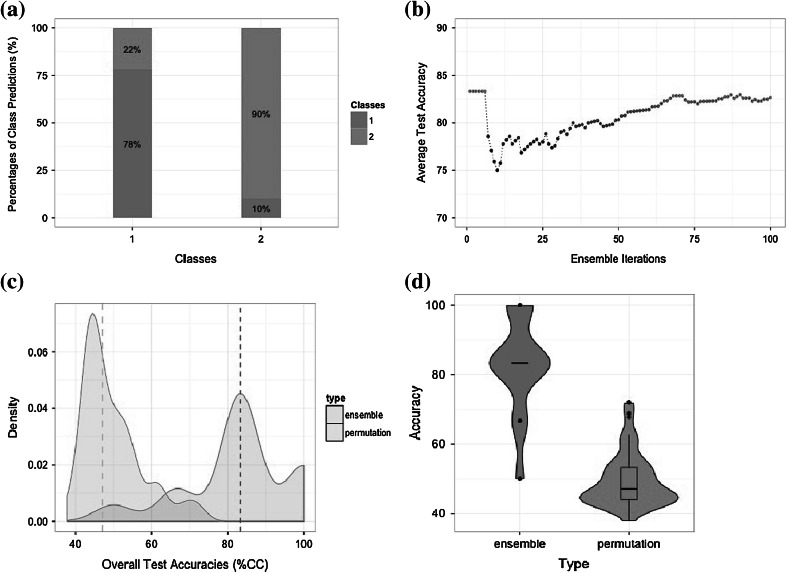
Performance metrics for dataset A, as calculated and plotted by *classyfire*. These show **a** classification accuracies per class; **b** average classification accuracy as a function of the number of SVMs in the ensemble; **c** a fused density plot that compares the ensemble and permutation distributions; **d** a fused *violin boxplot*, which combines the advantages of a *boxplot *and of a density shape plot, for a straightforward comparison of the ensemble and permutation distributions

The overall classification accuracy obtained for dataset A was equal to 82.7 %. A breakdown of the classification results by class is shown in Fig. 2a. Figure 2b depicts the overall classification accuracy as a function of the number of SVMs in the ensemble, which shows that it stabilises once the ensemble passes 75 classifiers, suggesting that the decision to use 100 classifiers was appropriate.

**Fig. 3:**
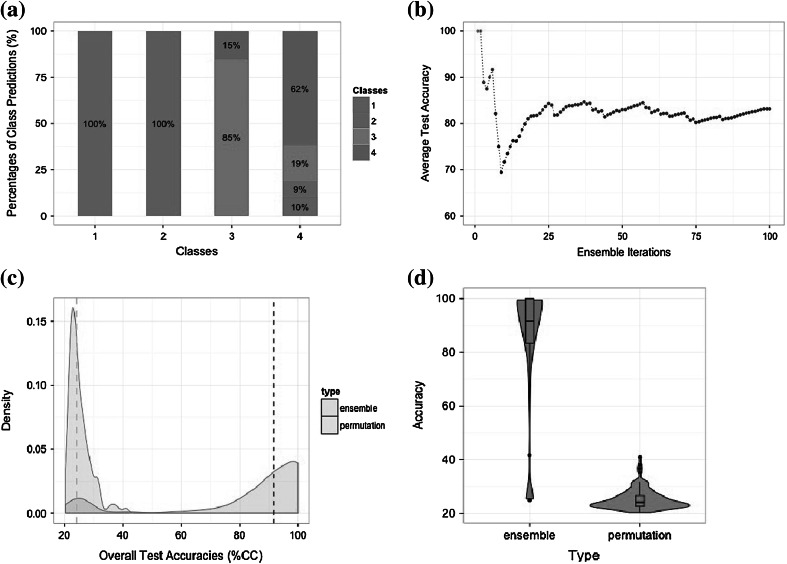
Performance metrics for dataset B, as calculated and plotted by *classyfire*. These show **a** classification accuracies per class; **b** average classification accuracy as a function of the number of SVMs in the ensemble; **c** fused density plot that compares the ensemble and permutation distributions; **d** a fused *violin boxplot*, which combines the advantages of a *boxplot* and of a density shape plot, for a straightforward comparison of the ensemble and permutation distributions

For dataset B, the overall classification accuracy was equal to 83.2 %, with most of the erroneous classification being related to class 4. The results are presented graphically in Fig. 3. The breakdown of the classification results by class (Fig. 3a) shows that samples belonging to classes 1 and 2 are always identified correctly, while samples from class 3 are occasionally (in 15 % of attempts) wrongly identified as class 4, and class 4 is the most difficult to predict, with frequent misassignments to other classes. Figure 3b depicts the overall classification accuracy as a function of the number of SVMs in the ensemble, and 100 again appears to be a reasonable number of classifiers to use in this case.

### Permutation testing results

Each permutation constitutes a single classification ensemble, which includes a predefined number of individual classifiers set by the user when using the *cfPermute()* function (by default equal to 100); each of these classifiers consists of 100 bootstrapping iterations (set by default) for the purposes of hyperparameter optimisation. The permutation tests were executed a total of 100 times for each dataset under study, which results in a total of one million iterations per dataset (since there are 100 classifiers per ensemble, each requiring 100 bootstrap iterations).

For both datasets under study, the non-permuted overall accuracies of the classification ensembles are well above the 95 % confidence intervals of the permutation distributions; indeed they are even greater than the 99 % confidence intervals leading to a greater confidence in our results. For instance, the non-permuted  %CC for the urine data (average test accuracy of the ensemble) is equal to 82.7 %, which is significantly higher than 53.3 % and 72.0 %, the values corresponding respectively to the upper 95 and 99 % confidence levels of the permuted distribution (Fig. 2c); the 95 % confidence interval of the distribution is retrieved using built-in *classyfire* functions as part of the “five number summary”, and can be graphically represented as in Figs. 2d and 3d. Similarly, in the case of the brain data, the non-permuted classification accuracy was equal to 83.2 % (Fig. 3c), well above the upper 95 and 99 % confidence levels, equal to 26.6 and 38.1 % respectively. In both instances, the *p* value was less than the significance level of 0.01, which gives us a strong indication about the statistical confidence of our results.

### Computational efficiency

**Fig. 4:**
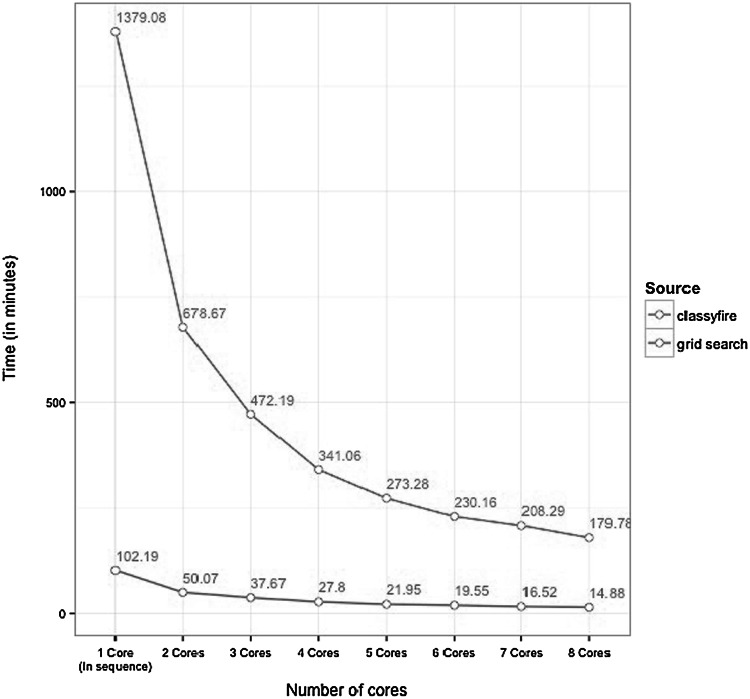
Execution time as a function of the number of processing cores used for training a classification ensemble for the urine dataset (dataset A), which consists of 100 independent classifiers, internally optimised using 100 bootstrap iterations (the default values of *cfBuild()* were used throughout). The analysis was executed on a dual CPU Intel Xeon X5660 at 2.8 GHz, which features 8 cores with 16 threads each and 32 GB RAM

In a direct comparison of the SVM optimisation algorithms, our heuristic method outperformed a traditional grid search by a factor of 13.5 when running on a single processing core. Training on multiple cores provides a speedup in proportion to the number of cores used. These results, obtained using the urine dataset, are shown in Fig. 4. Permutation testing was not included in this benchmarking experiment, but the processing required is directly proportional to the number of permutations, so execution times for a 100 iteration permutation test can be extrapolated to approximately 24 h for our heuristic method versus 12 days for the grid search.

## Discussion

By speeding up the SVM optimisation by more than an order of magnitude we have been able to produce a robust and easy to use multivariate classification package for R. While every effort has been made to ensure that the package produces high performance classification models, evaluated using the most accurate performance metrics available, there are of course limitations to what can be achieved with a given dataset. The experimental design used to generate the dataset is key. In particular, the generic applicability of the classification ensemble will be determined by the number of samples available, and how well those samples represent the biological phenomena under study. If variance observed in the real world is not represented in the data used to train and test the classification models then the performance reported by *classyfire* is unlikely to be achieved in real world application. This mistake is commonly made in clinical case/control studies where control samples are only taken from healthy volunteers, not from individuals with other diseases.

The current implementation of *classyfire* is solely focused on the optimisation of RBF SVMs with bootstrapping. As part of future developments, the application of the package could be extended to support different types of SVMs (e.g. polynomial kernel) as well as different types of classifiers.

## Conclusions

We have produced an easy to use R package that implements current best practice in the training and evaluation of models for recognising samples from analytical data acquired. Specifically, the package allows the user to build high performance ensembles of SVM classifiers and thoroughly evaluate and visualise the ensemble’s classification ability using bootstrapping and permutation testing. This has been made possible by developing a novel SVM optimisation strategy that reduces the time needed to execute this process by more than an order of magnitude. The package’s support for parallel processing enables execution time to be reduced even further, roughly as a function of the number of available processor cores. Our aim in releasing this package is to help increase the uptake of best practice by making our robust training and evaluation workflow available to biological researchers who may previously have been unable to do this due to lack of time or expertise.

## Abbreviations

ANN: Artificial neural network
%CC: Percent correctly classified
CPU: Central processing unit
LDA: Linear discriminant analysis
NMR: Nuclear magnetic resonance
PLS-DA: Partial least squares discriminant analysis
RBF: Radial basis function
SVM: Support vector machine
